# Sensory Description and Consumer Hedonic Perception of Ultra-High Temperature (UHT) Milk

**DOI:** 10.3390/foods11091350

**Published:** 2022-05-06

**Authors:** Yufang Su, Houyin Wang, Ziyan Wu, Lei Zhao, Wenqiang Huang, Bolin Shi, Jian He, Sisi Wang, Kui Zhong

**Affiliations:** 1 Inner Mongolia Yili Industrial Group Co., Ltd., Hohhot 010110, China; suyufang@yili.com (Y.S.); wuziyan@yili.com (Z.W.); ytnhuang@yili.com (W.H.); hejian@yili.com (J.H.); 2 Food and Agriculture Standardization Institute, China National Institute of Standardization, Beijing 100191, China; wanghy@cnis.ac.cn (H.W.); zhaolei@cnis.ac.cn (L.Z.); shibl@cnis.ac.cn (B.S.); wangss@cnis.ac.cn (S.W.)

**Keywords:** ultra-high temperature (UHT) milk, quantitative sensory description, consumer hedonic perception, partial least squares regression (PLSR) analysis

## Abstract

Sensory characteristics of products play an essential role on the consumer’ s acceptability, preference and consuming behavior choice. The sensory profiles and consumer hedonic perception for 14 UHT milk products using sensory quantitatively descriptive analysis and a 9-point hedonic scale were investigated in this study. There were significant differences in the sensory attributes intensity and liking scores among the organic whole milk, ordinary whole milk, low-fat milk, and skimmed milk (*p* < 0.05). Skimmed milk samples had lowest intensity scores of typical milk aroma, taste flavor and texture attributes, as well as had the lowest overall liking scores. Whole milk samples had higher sensory intensity scores than low-fat milk samples, even though no significant differences of overall liking scores were observed between whole milk and low-fat milk. Furthermore, the relationship between the sensory attribute and overall liking was demonstrated according to correlation analysis and partial least squares regression (PLSR) analysis. Overall liking increased significantly with the increasing of sweet, after milk aroma, protein-like, mellow and thick, while decreased significantly with the enhancement of cowy, cooked and whey (*p* < 0.05). These findings presented a potential strategy for identifying the key sensory attributes responsible for liking score differences among different kinds of UHT milk products.

## 1. Introduction

Bovine milk has long been considered as an important element in a healthy and balanced diet and a reliable source of nutrition for children and adults [1,2]. Bovine whole milk contains nutrients suitable for humans in a natural form, such as proteins, polyunsaturated fatty acids (FAs), vitamins, and minerals, rendering it one of the healthiest whole foods and the potential categories of resources for providing functional food products [3,4]. Past decades have seen a soar in China’s dairy production and consumption. China is the world’s second-largest dairy market after the US, accounting for nearly 28 million tons of products consumed in 2020. As suggested by the new Dietary Guidelines for Chinese Residents, an individual should consume more than 300 g of milk daily [5]. Ultra-high temperature (UHT) milk is a commercial dairy product that is sterilized at ≥135 °C for a few seconds [6]. Compared to traditional pasteurization, this process can obtain a product with an extended shelf-life of several months at ambient temperature [7,8]. Meanwhile, UHT milk reserves most nutrients and vitamins, making it essential for human consumption, and is driving the global market of dairy products. UHT milk products currently represent the largest category in the Chinese dairy market. Regarding fat content, UHT milk products are divided into three categories, including whole milk (fat content > 3%), low-fat milk (1.0~2.0%), and skimmed milk (fat content < 0.5%) [9]. In recent years, an increasing number of consumers are choosing low-fat and skimmed milk products for their health advantages, such as people with cardiovascular disease or obesity-related disorders [10]. Therefore, low-fat and skimmed milk are often preferred by consumers due to the loss of fat [11]. In a milk consumption and milk attitude survey of Brewer et al. [12], 82% of women chose the skim or reduced fat as the milk they most frequently consumed, although actually preferred whole milk having desirable sensory attributes.

Sensory characteristics is a vital quality of food products, playing a crucial role in product expectation, as well as consumers’ choice, purchase, preference and acceptability [13,14,15]. Therefore, it is necessary to understand the importance of sensory evaluation to assist the quality control of raw milk materials and consumption of varied dairy product categories. The main components in milk products determine the sensory profile [16]. For example, the role of milk fat has been scientifically described and it plays a vital role in the typical sensory characteristics (taste, aroma, flavor and texture) of milk products [17,18]. Milk contains numerous lipids, such as triacylglycerols, diacylglycerides, saturated/polyunsaturated fatty acids, and phospholipids, and the loss of fat could lead to the poor flavor of milk products [11]. Compared with UHT whole milk, the UHT skimmed milk had weaker intensities of sensory attributes (aroma properties) and lower contents of lipid-oxidized flavor compounds, including hexanal and 2-heptanone [19]. Previous reports have shown the influence of milk fat content on flavor release in milk and dairy products, and indicated that the fat content in milk was positively correlated with the stability of the entire system and product preference [11,20]. Currently, most studies involving the main components of milk products have focused on the change of volatile substance, product stability during shelf-life or flavor improvement technology of skimmed milk [11,18,21,22]. Few reports are available involving sensory evaluation and hedonic test of varied milk categories and assessing the relationship between sensory attributes and preference scores.

Therefore, this study focused on (1) establishing the sensory profiles of 14 types of UHT milk products, (2) determining the consumer preference differences of these milk products, and (3) investigating the relationship between the sensory attribute intensity and consumer preference scores. It further identified the key sensory attributes of UHT bovine milk contributing to the preference scores.

## 2. Materials and Methods

### 2.1. Materials

Fourteen UHT milk products were obtained from local Chinese supermarkets. Information regarding the main components is shown in Table 1, including the fat, protein, carbohydrate, and sodium content, and was derived from the nutritional composition labels on the product packaging. Next, the 14 milk products were divided into four categories according to the fat content (two organic whole milk, seven non-organic whole milk, two low-fat milk, and three skimmed milk products). Only one UHT milk product (P7) originated in another country, with a production date of October 2020. The remaining 13 UHT milk products were domestically manufactured, with production dates of January 2021. This study was conducted in March 2021 and lasted one month.

### 2.2. Sample Preparation

Fresh milk samples were prepared before each sensory evaluation or consumer hedonic test. The tests were conducted within 24 h of milk samples preparation. Next, individual milk samples of 15 mL each were placed in separate 50 mL transparent tasting cups with lids and labeled with random three-digit numbers. Sample presentation order to the panelists was randomized.

### 2.3. Sensory Panel

The sensory assessors were selected, trained, and monitored according to the ISO 8586 standards [23]. The sensory panel consisted of 12 experienced assessors (6 females and 6 males, mean age: 28.4 ± 2.9) selected from a dairy milk products sensory analysis laboratory and displaying excellent sensory sensitivity. They were required to be healthy, not use drugs or smoke, and be free of chronic diseases, such as hypertension or heart disease. Furthermore, all participants were required not to have experienced any cold symptoms for at least one week before the sensory evaluation and had to have sensory evaluation experience of dairy products. The training schedule of the sensory panel included the identification and description of the milky flavor and the procedures involving the response scale. The sensory evaluation only commenced when the evaluation panel displayed excellent repeatability and consistency.

### 2.4. Sensory Evaluation

The conditions of the sensory evaluation environment, including the area of sample preparation, sensory evaluation, and concentrative discussion, met the requirements of the ISO 8589 standard [24]. The sensory evaluation area was kept in an adequately air-conditioned environment, and the temperature in the booths was controlled at about 25 °C.

The sensory profiles of the milk samples were established according to the ISO 13299 standard [25]. Here, 12 selected assessors were required to generate the sensory descriptors in a round table discussion under the direction of an experienced panel leader. The descriptors of the sensory attributes were developed according to the ISO 5492 standard [26]. The definition (Table 2) and corresponding reference samples of each sensory attribute were developed via a panel discussion. Furthermore, the sensory attribute intensity of the 14 milk samples was evaluated using a 15-point line scale, denoting an interval quantitative response scale ranging from 0 (none) to 15 (extremely strong), signifying low to high intensity [27]. The samples were labeled with random three-digit numbers and arbitrarily presented to the assessors. The 14 milk samples were evaluated over six sessions, presenting four or five samples per session to prevent sensory and mental fatigue. The assessors were allowed a short break (5 min) between sessions, and each sample was presented at least twice. Plain crackers and purified drinking water were provided as palate cleansers. The next sample was only evaluated when the residual taste was cleared from the mouth. Finally, the sensory profiles were established via the qualitative and quantitative analyses of the 15 sensory attributes. This sensory study was reviewed and approved by the Ethics Committee of Tsinghua University and informed consent was obtained from each subject prior to their participation in the study.

### 2.5. Consumer Hedonic Perception

Since the consumer hedonic tests in this study were conducted in controlled areas, consumer recruitment occurred according to the ISO 11136 standard [28]. Here, 61 adult consumers (25F36M, mean age: 25.4 ± 2.2) were recruited and required to consume milk at least twice a week. Then, the acceptability of the consumers toward the UHT milk samples was evaluated using a 9-point hedonic scale (1 = dislike extremely, 2 = dislike very much, 3 = dislike, 4 = dislike slightly, 5 = neither like nor dislike, 6 = like slightly, 7 = like, 8 = like very much, 9 = like extremely). AppSense V5.0 software (Yinghuali Technology Co., Ltd., Beijing, China) was used to conduct the test and collect consumer responses. Fourteen milk samples were tested over 3 sessions, presented one by one and in random order. The consumers were requested to have a short break (5 min) between sessions, and each sample was tested at least twice. Soda biscuits and purified drinking water were provided as palate cleansers. The next sample was only evaluated when the residual taste was cleared from the mouth. The sensory evaluation area was kept in an adequately air-conditioned environment, and the room temperature was controlled at about 25 °C.

### 2.6. Statistical Analysis

One-way analysis of variance (ANOVA) and Tukey test were used to identify the differences between the sensory attribute data and the overall liking data at significance level of 0.05 regarding the 14 UHT milk samples. ANOVA was performed using IBM SPSS Statistics 22 software (SPSS Inc., Chicago, IL, USA). The radar chart and cluster heatmap of the sensory attribute description in different UHT milk products were created using Origin 2018 software (OriginLab Corporation, Northampton, MA, USA). Partial least squares (PLS) analysis was employed to investigate the relationship between the sensory attributes and overall liking values (OL) using SIMCA 14.0 software (Umetrics Inc., Malmo, Sweden).

## 3. Results

### 3.1. Sensory Descriptors Generation

**Figure 1:**
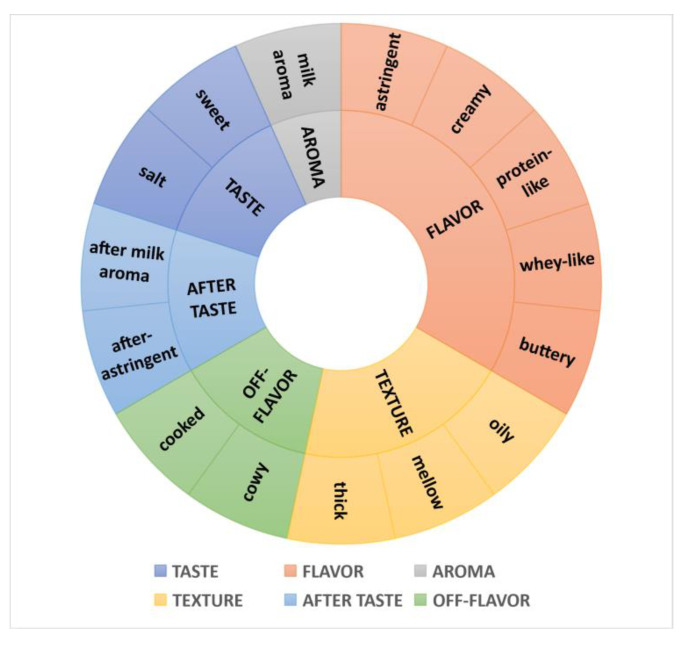
The sensory flavor wheel of UHT milk.

The sensory panel evaluated the 14 UHT milk samples and developed the sensory attribute descriptors, which were further identified and selected via a multidimensional approach according to the ISO 11035 standard [29]. Ultimately, 15 sensory descriptors were determined in six dimensions to establish the sensory profile of UHT milk products. Figure 1 demonstrate the 15 sensory descriptors of the UHT milk samples, including two taste descriptors, namely sweet (SW) and salty (SA), five flavor descriptors, namely protein-like (PRO), creamy (CR), whey-like (WH), buttery (BU), and astringent (AS), one aroma descriptor, namely a milky aroma (MA), three textural descriptors, namely thick (TH), mellow (ME), and oily (OIL), two after taste descriptors, namely after-milky aroma (AMA) and after-astringency (AAS), and two off-flavor descriptors, namely cooked (CO) and cowy (COW).

Milk aroma, milk flavor, sweet and residual mouthfeel were the typical sensory descriptors of liquid milk [11,13,18,27]. Richardson-Harman et al. [20] and Jervis et al. [30] showed creaminess flavor descriptors of high fat dairy products, including the cream aroma, butter aroma, vanilla flavor, and the texture characteristics of mouthfeel (slipperiness, higher viscosity, etc.). Tong et al. [11] also reported that skim milk was characterized by a low salty taste, which was consistent with the result of this study. Additionally, the descriptors of sour and umami were also generated in their study, but these two descriptors were not produced by our sensory assessors in this study. The off-flavors of liquid milk are directly responsible for product rejection by the consumer [13]. Cooked was the thermally derived off-flavor descriptor in milk, and cowy was the off-flavor descriptor associated with the raw milk material [13].

### 3.2. Sensory Quantitative Descriptive Analysis

**Figure 2:**
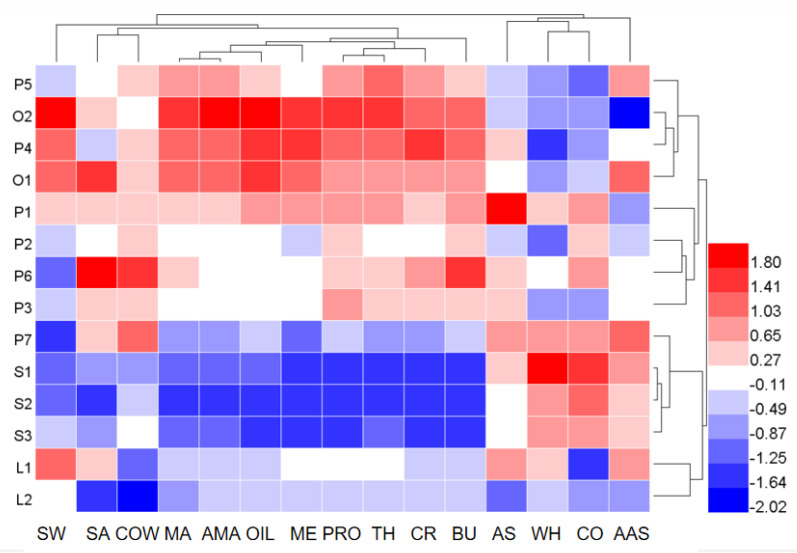
Concentration heatmap of sensory attributes of the 14 UHT milk samples.

**Figure 3:**
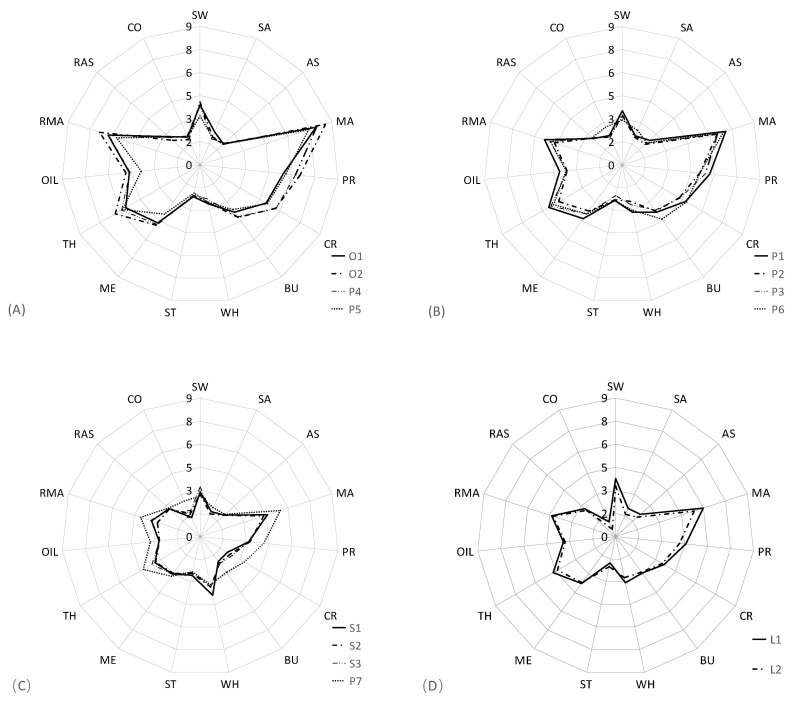
The sensory profile description of 4 different categories of UHT milk samples (n = 12). (**A**) Cluster 1, (**B**) cluster 2, (**C**) cluster 3, and (**D**) cluster 4.

The concentration heatmap of sensory attribute intensity of 14 UHT milk samples is shown in Figure 2, which illustrated the sensory attribute differences of varied milk samples. The color coding was graded according to the scale from blue to red, with the relative intensity increasing from low (blue) to high (red) [31]. The 14 milk samples were divided into four clusters (Figure 2), and the quantitative sensory profiles of the four different clusters were further described using radar charts (Figure 3). The quantitative descriptive analysis results indicated that the UHT milk samples displayed similar sensory profiles, with the typical sensory characteristics including milk aroma (MA, AMA), milk flavor (PRO, CR, BU), texture (TH, ME, OIL) and basic taste (SW), which exhibited high-intensity scores, whereas they presented lower scores of off-flavor (CO, COW, AS and AAS), SA and WH. This result was consistent with the previous study [11,17].

Cluster 1 included two organic whole milk samples (O1 and O2) and two non-organic whole milk samples (P4 and P5). This group displayed highest intensity scores of aromas (MA and AMA), textural (TH, ME, and OIL), typical milk flavor (PRO, CR, and BU), and taste (SW), and low intensity scores of off-flavor (AS and CO) and WH, while a medium intensity score was evident in the COW (Figure 3A). The ANOVA analysis indicated that 8 of 15 sensory attributes (SW, MA, PRO, CR, BU, TH, OIL, AMA) showed significant differences among four milk samples (*p* < 0.05). Cluster 2 included four non-organic whole milk samples (P1, P2, P3, and P6), displaying the significant lower scores for milk aroma and flavor, texture and SW compared with cluster 1 (Figure 3B). Furthermore, P6 exhibited significant higher scores for COW, SA and BU than the other three non-organic whole milk samples (*p* < 0.05).

Cluster 3 consisted of the three skimmed milk and whole milk samples (P7), displaying the lowest scores for the milk aroma and flavor, texture and sweet, the highest scores for the WH and CO, and medium scores of COW (Figure 3C). Significant differences were apparent between the scores of CR, SA, and WE of the three skimmed milk samples and P7 (*p* < 0.05). Cluster 4 consisted of two low-fat milk samples, displaying the lower scores for the milk aroma, milk flavor and texture compared with the whole milk samples (Figure 3D), whereas the milk samples in cluster 3 had higher scores on sweet and lowest scores on off-flavor (COW and CO). Significant differences were evident between the scores for SW, SA, and MA scores of the L1 and L2 samples (*p* < 0.05).

The sensory characteristics were strongly influenced by the primary properties of product composition [16,32]. Milk fat played a key role in the sensory characteristics of milk products, and the higher fat content promoted the enhancement of typical milk traits of aroma, flavor, taste and texture attributes [18,33]. The result in this study also showed a similar conclusion, and whole milk samples had the highest scores of typical sensory attributes, followed by low-fat milk samples and finally the skimmed milk samples. Furthermore, the whole milk samples in cluster 1 had higher scores of sensory attributes than the whole milk samples in cluster 2, which were also the ones with higher protein, fat, and carbohydrate content [34,35]. It was also observed that skimmed milk samples had highest scores of cooked and lowest scores of cowy, this was consistent with those reported by other studies [11,13]. Heat treatments, particularly UHT processing, could enhance the intensity of thermally derived off-flavor attribute, such as cooked [36]. The low contents of milk fat could reduce the formation of cowy in the milk production. Furthermore, the P7 whole milk samples exhibited similar intensity scores to the skimmed milk samples, which was significantly lower than the other whole milk samples. This is assumed to be related to the country of origin of the milk and its shelf-life. P7 represented the imported milk, which had a shelf-life of six months, while that of the remaining 13 samples was only about two months.

### 3.3. Consumer Hedonic Tests

**Figure 4:**
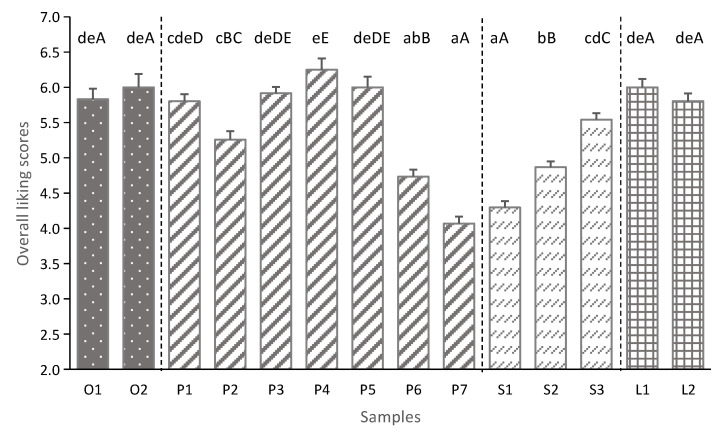
The overall liking scores of 14 UHT milk samples. (n = 61, values are mean ± SE). Repeated measure ANOVA was used in this study. Lower case indicates the statistical differences (*p* < 0.05) between 14 UHT milk samples; upper case indicates the statistical differences (*p* < 0.05) between the different UHT milk samples in the same milk product category (organic whole milk, non-organic whole milk, low-fat milk and skimmed mil).

The result of consumer preference was shown in Figure 4. The overall liking scores of the 14 UHT milk samples ranged from 4.07 to 6.25 in a 9-point hedonic scale, that it corresponds from ‘dislike slightly’ to ‘like slightly’. In general, a score higher than 5 indicates that consumer like the product [27]. In this study, ten of the 14 milk samples had good degree of liking scores (5.54 to 6.25), indicating that most of the milk samples were favored by the consumers. There were significant differences in overall liking scores of 14 UHT milk samples (*p* < 0.05). No significant differences were evident between the liking scores of low-fat milk samples, organic and most non-organic whole milk according to ANOVA (*p* > 0.05). The skimmed milk samples (S1 and S2) and some whole milk samples (P6 and P7) had the lowest liking scores (<5). Besides, the differences of liking scores in the same milk category were further investigated, and there was a significant difference in the liking scores of the three skimmed milk samples and seven non-organic whole milk samples, which were S1 < S2 < S3 and P7 < P6 = P2 < P1 = P3 = P5 < P2, respectively (*p* < 0.05).

This result was consistent with the previous study, that the most consumers showed lowest liking score to the skimmed milk, and non-significant difference was observed between whole fat and semi-skimmed preference [14,33]. The content of fat in the milk product has been shown to be associated with the consumer’ s preference and flavor perception [13,37]. McCarthy et al. [18] also pointed out that the consumer’s preference for whole milk was significantly higher compared with skimmed milk (*p* < 0.05). Besides fat, the other main components in milk products also determine the consumer preference and behaviors to products [16,38]. In this study, the liking score of skimmed milk S3 (5.54) was significantly higher than other two skimmed milk S1 and S2, which has been considered to be related to the higher contents of protein and carbohydrate in S3 sample (Table 1). The raw milk materials of these three skimmed milk samples came from different milk source areas, which was considered to be the reason for the different contents of the main constituents.

### 3.4. The Relationship between Sensory Attributes and Overall Liking

#### 3.4.1. Correlation Analysis

Significant differences of sensory attributes and overall liking (OL) were observed in the same milk category in this study. Previous study showed that the sensory characteristics of the liquid milk strongly influence consumer’s acceptability and preference [14]. Therefore, the relationship between the sensory attribute and overall liking was investigated in this study.

**Figure 5:**
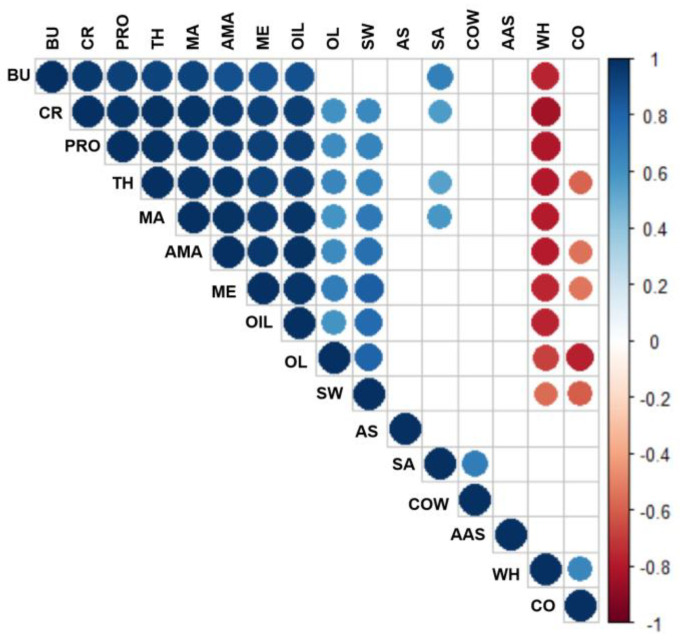
Correlation analysis of overall liking (OL) scores to sensory attributes in UHT milk samples. The blue and red dots represented significant positive and significant negative correlations, respectively (*p* < 0.05). The blank denoted no significant correlation, while the color of the dots became darker as the correlation coefficient increased.

Figure 5 showed the partial correlations between the overall liking score to sensory attribute. The blue and red dots represented significant positive and significant negative correlations, respectively (*p* < 0.05). The blank denoted no significant correlation, while the color of the dots became darker as the correlation coefficient increased. The OL score was significantly correlated with most of the sensory attributes, suggesting that the consumer overall liking was strongly influenced by the sensory attributes of the UHT milk samples. Furthermore, the OL scores exhibited a significant positive correlation with all aroma and textural attributes, most flavor attributes (PRO, CR, and OIL), and SW (*p* < 0.05). No significant correlation was evident between the OL score and sensory attributes of AS, AAS, COW, and SA, while a significant negative correlation was apparent between the OL scores and attributes of CO and WH, respectively. This result was consistent with the previous reports, Richardson-Harman et al. [20] and Jervis et al. [30] indicated the effects of fat contents and creaminess perception on the overall liking in dairy products, respectively.

#### 3.4.2. PLSR Analysis

**Figure 6:**
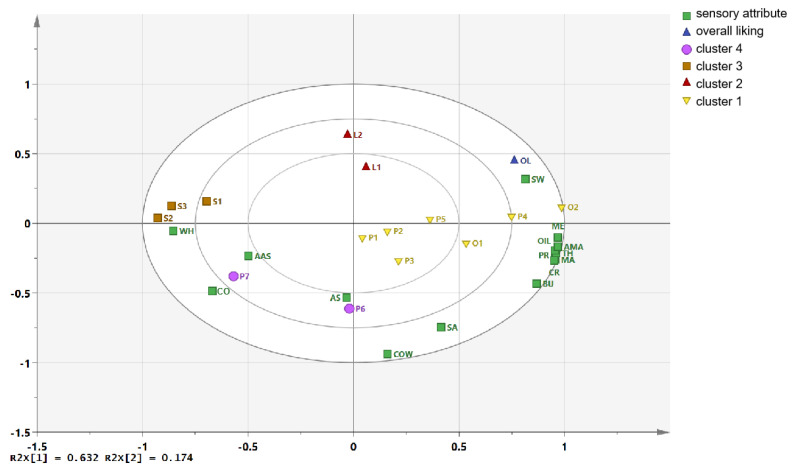
PLSR bi-plot diagram between sensory attribute and overall liking (OL) of the 14 UHT milk samples.

The correlations between sensory attribute and overall liking were further explained by the PLSR analysis. PLSR was used to determine the relationship between independent and dependent variables (numerical variables) [27], and was used in this study to formulate mathematical model for establishing the association between the sensory attributes and OL scores of UHT milk samples. As shown in Figure 6, PLSR provided a binary factor model that explained 80.7% of the *x*-variance (sensory attribute) and 84.4% of the *y*-variance (overall liking score). Most of the sensory attribute data and overall liking data of the UHT milk samples were located between the internal and external ellipses at *r*^2^ = 0.5 and 1.0, respectively, indicating that they were well explained by the PLSR model [10].

T1 and T2 explained 63.2% and 17.4% of the total variance, respectively. Therefore, the first two dimensions defined 80.6% of the total variations. The OL appeared on the right side along the *x*-axis, displaying a strong positive correlation with the taste (SW), aroma (MA and AMA), textural (TH, ME, and OIL), and most flavor attributes (PRO and CR). Some off-flavor attributes, such as CO, WH, and AAS, were located on the left side of the *x*-axis, indicating a strong negative correlation with the OL. Furthermore, the OL exhibited a weak correlation with COW, AS, and SA, which was consistent with the correlation analysis and previous reports [11,18,20,30].

The 14 UHT milk samples were divided into four clusters by hierarchical clustering analysis (Figure 6). There were significant differences in sensory attribute and liking score among different clusters. Cluster 1 consisted of two organic whole milk samples (O1 and O2) and five ordinary whole milk samples (P1–P5). These seven samples appeared on the right side of the *x*-axis, and displaying the higher liking scores and a strong positive correlation with SW, milk aroma and flavor (MA, AMA, BU, PRO, CR) and texture attribute. These were the key sensory attributes that contribute to high liking scores of liquid milk, which were consistent with previous studies [37]. Cluster 2 consisted of two low-fat milk samples (L1 and L2), which were located at the top of the *y*-axis. This cluster showed no significant difference in liking score compared with cluster 1, while they had significant differences in the sensory attribute. It showed weak correlation with milk aroma, flavor and texture attribute, while showing a strongly negative correlation with COW and ST. Both cluster 3 and cluster 4 had the lowest liking scores.

Cluster 3 (three skimmed milk samples) was evident on the leftmost side of the *x*-axis, denoting opposite sensory and preference characteristics than cluster 1. These three milk samples demonstrated a strong negative correlation with the typical sensory attributes of SW, milk aroma, flavor and texture attribute, a positive correlation with WH and a medium degree correlation with CO and AAS. Milk fat is known to have the most direct contribution to sensory aromas and flavor perception [18,30]. Therefore, skimmed milk samples had weak milk aroma and flavor for the lack of fat contents, which has been considered to be the one of most important reasons for the lowest OL scores. P6 and P7 belonged to cluster 4, which appeared on the bottom left of the zero-axis, displaying a strong positive correlation with off-taste (ST and COW), AS, and AAS, which were considered to be the main sensory characteristics leading to the lower liking scores.

**Figure 7:**
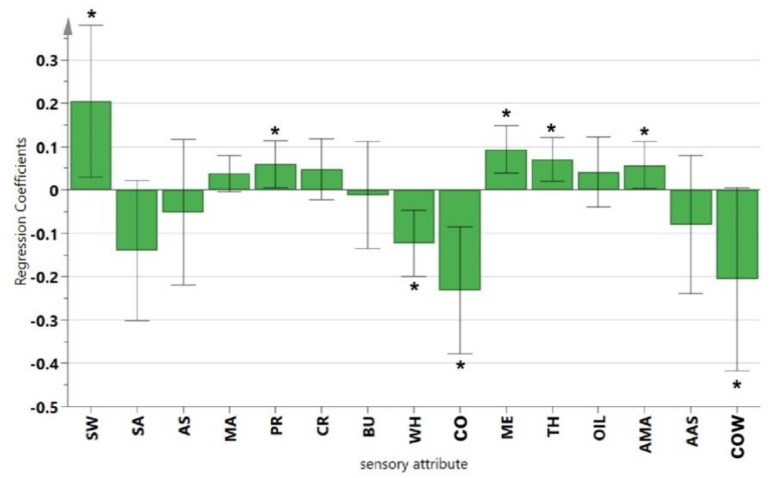
PLS regression coefficients of the UHT milk samples. *: indicates the significant effect (α = 0.05) of this sensory attibute on the OL value.

The model quality exceeded 0.6 (Q^2^ = 0.703), and the difference between R^2^ and Q^2^ was below 0.3, indicating that the PLSR model explained the data well [10]. Moreover, The PLSR coefficients and their significance were shown in Figure 7. It could be seen that the OL increased significantly with the increasing of one taste attribute (SW), one after-taste attribute (AMA), one flavor attribute (PR) and two textural attributes (ME and TH) (*p* < 0.05). In contrast, the OL decreased significantly when the two off-flavor attributes (COW and CO) and one flavor attribute (WH) increased (*p* < 0.05). This result was consistent with the correlation analysis of the above.

## 4. Conclusions

This study investigated the sensory profiles and consumer hedonic perception for 14 UHT milk products. There were significant differences in the sensory attributes intensity and liking scores among the organic whole milk, ordinary whole milk, low-fat milk, and skimmed milk (*p* < 0.05). Skimmed milk samples had lowest scores of typical milk aroma, taste flavor and texture attributes, as well as had the lowest overall liking scores (*p* < 0.05). No significant differences of overall liking scores were observed between the organic whole milk, most non-organic whole milk and low-fat milk (*p* > 0.05). Furthermore, different milk samples in the same milk category had different sensory attributes intensities and liking scores, including the whole milk and skimmed milk products. This has been considered to be related to the varied contents of the main components in milk, such as fat, protein, etc. The relationship between the sensory attribute and overall liking was demonstrated according to correlation analysis and PLSR analysis. Overall, liking increased significantly with the increasing of sweet, after milk aroma, protein-like, mellow and thick, while it decreased significantly with the enhancement of cowy, cooked and whey (*p* < 0.05). These findings presented a potential strategy for identifying the key sensory attributes responsible for liking score differences among different kinds of milk products and improving the sensory quality and acceptability of UHT milk products.

## Figures and Tables

**Table 1:** The main components information of 14 UHT products (g/100 mL).

Category	Code	Fat	Protein	Carbohydrate	Na^+^
Organic whole milk	O1	4.6	3.8	5.5	60 × 10^−3^
	O2	4.6	3.8	5.5	60 × 10^−3^
Non-organic whole milk	P1	3.8	3.2	4.8	53 × 10^−3^
	P2	3.8	3.2	4.8	53 × 10^−3^
	P3	3.8	3.2	4.8	53 × 10^−3^
	P4	4.4	3.6	5.0	58 × 10^−3^
	P5	4.4	3.6	5.0	58 × 10^−3^
	P6	3.6	3.2	4.5	50 × 10^−3^
	P7	3.5	3.3	4.8	45 × 10^−3^
Low-fat milk	L1	1.4	3.6	5.1	58 × 10^−3^
	L2	1.3	3.6	5.1	67 × 10^−3^
Skimmed milk	S1	0	3.2	5.0	53 × 10^−3^
	S2	0	3.2	5.0	53 × 10^−3^
	S3	0	3.8	5.5	60 × 10^−3^

**Table 2:** Sensory descriptors and their definitions for UHT milk.

Sensory Attribute	Descriptor	Definition
Taste	Sweet	basic taste produced by sugar solution
	Salt	basic taste produced by sodium chloride solution
Flavor	protein-like	the flavor like protein solution
	creamy	the flavor like cream
	whey-like	the flavor like whey solution
	buttery	the flavor like butter
	astringent	a complex sensation, accompanied by shrinking of the skin or mucosal surface in the mouth
Aroma	milky aroma	the normal odors of milk, the aroma is natural and without an off-smell
Texture	thick	a mechanical textural attribute relating to flow resistance
	mellow	a textural attribute relating to sensation of fullness and richness
	oily	a textural attribute relating to fat (quality and quantity) sensation
After-taste	after-milky aroma	the sensation of milk aroma that occurs after the elimination of the product
	after-astringency	astringency sensation that occurs after the elimination of the product
Off-flavor	cooked	sulfurous flavor relating to cooked milk products
	cowy	specific flavor relating to the smell of cow or cow-beef, the flavor is usually unpleasant

## Data Availability

Data is contained within the article.
